# On the Ancestral UDP-Glucose Pyrophosphorylase Activity of GalF from *Escherichia coli*

**DOI:** 10.3389/fmicb.2015.01253

**Published:** 2015-11-13

**Authors:** Ana C. Ebrecht, Agnieszka M. Orlof, Natalia Sasoni, Carlos M. Figueroa, Alberto A. Iglesias, Miguel A. Ballicora

**Affiliations:** ^1^Instituto de Agrobiotecnología del Litoral, Universidad Nacional del Litoral – Consejo Nacional de Investigaciones Científicas y Técnicas – Centro Científico Tecnológico CONICET Santa FeSanta Fe, Argentina; ^2^Department of Chemistry and Biochemistry, Loyola University Chicago, ChicagoIL, USA

**Keywords:** enzyme resurrection, enzyme evolution, galactose metabolism, enzyme inactivation, catalytic residues, nucleotidyltransferase

## Abstract

In bacteria, UDP-glucose is a central intermediate in carbohydrate metabolism. The enzyme responsible for its synthesis is encoded by the *galU* gene and its deletion generates cells unable to ferment galactose. In some bacteria, there is a second gene, *galF*, encoding for a protein with high sequence identity to GalU. However, the role of GalF has been contradictory regarding its catalytic capability and not well understood. In this work we show that GalF derives from a catalytic (UDP-glucose pyrophosphorylase) ancestor, but its activity is very low compared to GalU. We demonstrated that GalF has some residual UDP-glucose pyrophosphorylase activity by *in vitro* and *in vivo* experiments in which the phenotype of a *galU*^-^ strain was reverted by the over-expression of GalF and its mutant. To demonstrate its evolutionary path of “enzyme inactivation” we enhanced the catalysis by mutagenesis and showed the importance of the quaternary structure. This study provides important information to understand the structural and functional evolutionary origin of the protein GalF in enteric bacteria.

## Introduction

The biosynthesis of glycoconjugates essentially depends on the availability of activated sugars. Leloir (1971) discovered the importance of nucleoside-diphosphate-sugars (NDP-sugars) in metabolism nearly 60 years ago after isolating UDP-glucose (UDP-Glc) from yeast. This molecule is employed in all organisms for glycosyl transfer reactions and as a precursor for various activated carbohydrates (Roeben et al., 2006). In bacteria, UDP-Glc has a number of important metabolic functions, including the biosynthesis of lypo- (Sandlin et al., 1995; Dean and Goldberg, 2002; Vilches et al., 2007) and exo-polysaccharides (Becker et al., 1998; Mollerach et al., 1998), capsule and membrane-derived oligosaccharides (Komeda et al., 1977; Sandlin et al., 1995; Bonofiglio et al., 2005), trehalose (Giaever et al., 1988; Boos et al., 1990; Padilla et al., 2004), and cellulose (Ross et al., 1991). The production of this key metabolite is mediated by UDP-Glc pyrophosphorylase (UDP-Glc PPase, UTP:glucose-1-phosphate uridylyltransferase, EC 2.7.7.9). This enzyme catalyzes, in an Mg^2+^-dependent reaction, the reversible formation of UDP-Glc and pyrophosphate (PPi) from glucose-1-phosphate (Glc-1P) and UTP (Roeben et al., 2006). It has been determined that this protein is encoded by the *galU* gene in prokaryotes (Weissborn et al., 1994; Boels et al., 2001; Bonofiglio et al., 2005; Vilches et al., 2007). The UDP-Glc PPase is a key enzyme in Glc anabolism in all organisms. Deletion or mutation of this gene in different bacteria generates cells that cannot ferment galactose (Gal) and fail to incorporate Gal and Glc into the bacterial cell membranes. This leads to an incomplete synthesis of lipopolysaccharides (LPS) in Gram-negative bacteria (Sundararajan et al., 1962; Sandlin et al., 1995; Dean and Goldberg, 2002; Vilches et al., 2007) and the incomplete formation of the polysaccharide capsule in Gram-positive bacteria (Sundararajan et al., 1962; Weissborn et al., 1994; Bonofiglio et al., 2005).

In enterobacteria, it has been identified a second gene named *galF*, which encodes for a protein homologous to GalU (Jiang et al., 1991; Klena and Schnaitman, 1993; Macpherson et al., 1994; Xiang et al., 1994; Yao and Valvano, 1994; Marolda and Valvano, 1995, 1996). GalF from *Escherichia coli* shares ∼36–58% identity with different prokaryotic UDP-Glc PPases (Marolda and Valvano, 1996). It has been suggested that GalF interacts with GalU (Marolda and Valvano, 1996) and regulates the intracellular levels of UDP-Glc (Nakae, 1971). Despite the presence of *galF*, the *E. coli* UDP-Glc PPase has been characterized as a homotetrameric enzyme consisting of four GalU subunits (Hossain et al., 1994; Weissborn et al., 1994). Studies at the molecular level are scarce to clearly establish a functional role (if any) for GalF at the present time. It has only been reported that GalF did not have detectable UDP-Glc PPase activity (Marolda and Valvano, 1996).

Here, we biochemically investigated the ancestral catalytic role of GalF and its evolutionary origin. We cloned the genes *galU* and *galF* from *E. coli* and after recombinant expression, purification, quaternary structure determination, and *in vivo* and kinetic studies we characterized a vestigial GalF activity. We explained the reasons for the activity loss and proceeded to partially revert it by mutagenesis. This study provides important clues to understand the structural and functional origin of the GalF protein in enteric bacteria. This seems to be one case of evolution by “enzyme inactivation” (Thornton, 2004).

## Materials and Methods

### Bacterial Strains and Media

*Escherichia coli* TOP10 (Invitrogen, Carlsbad, CA, USA) were used for cloning procedures and plasmid maintenance. Protein expression was carried out with *E. coli* BL21 Star (DE3; Invitrogen). *E. coli* FF4001 (Harding et al., 1993) were used for complementation assays. Cells were grown in LB medium supplemented with kanamycin (50 μg/ml) when necessary.

### Cloning of *galU* and *galF* Genes

The genes coding for GalU (*galU*) and GalF (*galF*) were amplified by PCR using *E. coli* K-12 genomic DNA as template and Phusion DNA polymerase (New England BioLabs, Ipswich, MA, USA). Supplementary Table S1 shows the specific oligonucleotide pairs (forward and reverse) used for cloning these genes. PCRs were performed with the following cycling parameters: one cycle of 5 min at 98°C, 30 cycles of 30 s at 98°C, 20 s at 50°C and 1 min at 72°C, followed by a final cycle of 5 min at 72°C. The amplified genes were cloned into the StrataClone vector (Stratagene, La Jolla, CA, USA) and their identities were confirmed by complete sequencing (University of Chicago CRC, Chicago, IL, USA). The *galF* and *galU* genes were subcloned into pET28c vector. With this strategy, GalF was expressed as a His-tag fusion protein and GalU as an untagged protein. Restriction sites used for each gene are specified in Supplementary Table S1. In addition, *galF* was subcloned into pET24a in order to obtain the recombinant protein without the tag.

For genetic complementation assays, the genes coding for GalU, GalF, and the mutant GalF_M15T/H16R_ were subcloned into pMAB5 vector (pGALU, pGALF, and pM15T/H16R, respectively). pMAB5 is an expression vector which uses IPTG (isopropyl β-D-1-thiogalactopyranoside) as inducer (Bejar et al., 2004).

### Site-Directed Mutagenesis

Site-directed mutagenesis was performed by PCR overlap extension (Sambrook and Russell, 2001) using Phusion DNA polymerase. Plasmids encoding the wild-type enzymes were used as templates. The sequence of each mutant was verified by double strand sequencing. Oligonucleotides used for mutagenesis are shown in Supplementary Table S1.

### Protein Expression and Purification

As a general procedure, 1 l of LB medium supplemented with 50 μg/ml kanamycin was inoculated with a 1/100 dilution of an overnight culture of transformed *E. coli* BL21 Star (DE3). Cells were grown at 37°C in an orbital shaker, at 200 rpm, until OD_600_ ∼0.6 was reached and then induced overnight with 0.4 mM IPTG at 25°C. Cells were harvested by centrifuging 15 min at 4°C and 5000 × *g*, and the pellet was frozen at –20°C until use.

To purify wild-type and mutant forms of GalU, as well as GalF from pET24a vector, cells were resuspended in *buffer A* [25 mM Tris-HCl pH 8.0, 5% (w/v) sucrose, 5 mM MgCl_2_, and 0.1 mM EDTA] and disrupted by sonication. The resultant suspension was centrifuged 15 min at 4°C and 10000 × *g* and the supernatant was loaded in 5 ml DEAE-Sepharose Fast Flow column (GE Healthcare, Piscataway, NJ, USA) previously equilibrated with *buffer A*. The column was washed with 10 bed volumes of *buffer A* and then with increasing concentrations of NaCl (50, 100, and 200 mM) in *buffer A* (five bed volumes each). The recombinant proteins were usually recovered in the fraction containing 200 mM NaCl.

Wild-type and mutants of His-tagged GalF were purified by pseudo-affinity chromatography. Cells were resuspended in *buffer B* [25 mM Tris-HCl pH 8.0, 300 mM NaCl, 10 mM imidazole and 5% (v/v) glycerol] and disrupted by sonication. The resultant suspension was centrifuged 15 min at 4°C and 10000 × *g* and the supernatant was loaded in a 1 ml His-Tag column (GE Healthcare) previously equilibrated with *buffer B*. The column was washed with 10 bed volumes of *buffer B* and then with increasing concentrations of imidazole (40, 80, and 160 mM) in *buffer B* (five bed volumes each). The recombinant proteins were usually recovered in the fraction containing 160 mM imidazole.

Active fractions were pooled, dialyzed to remove salts and supplemented with 10% (v/v) glycerol. Samples were conveniently fractionated and stored at –80°C. Under these conditions the recombinant enzymes were stable for at least 6 months. Protein purify was evaluated by densitometry with the program ImageJ (Schneider et al., 2012).

### Protein Methods

Protein concentration was determined by the Bradford (1976) method, using bovine serum albumin (BSA) as a standard. Protein electrophoresis under denaturing conditions (SDS-PAGE) was carried out on discontinuous 12% polyacrylamide gels, as previously described (Laemmli, 1970).

### Enzyme Activity Assays

UDP-Glc PPase activity was determined at 37°C in the UDP-Glc synthesis direction, by following the formation of Pi (after hydrolysis of PPi by inorganic pyrophosphatase) by the colorimetric method previously described (Fusari et al., 2006). Unless otherwise specified, reaction mixtures contained 50 mM MOPS pH 8.0, 10 mM MgCl_2_, 2 mM UTP, 0.2 mg/ml BSA, 0.5 U/ml yeast inorganic pyrophosphatase, and enzyme in an appropriate dilution. Assays were initiated by the addition of 2 mM Glc-1P in a total volume of 50 μl. Reaction mixtures were incubated for 10 min at 37°C and terminated by adding the color reagent (Malachite Green). The complex formed with the released Pi was measured at 630 nm with a microplate reader (Thermo Electron Corporation, Vantaa, Finland).

### Kinetic Studies

Saturation curves were performed by assaying enzyme activity with varying concentrations of one substrate while keeping saturating levels of the other. Experimental data were plotted as enzyme activity (U/mg) *versus* substrate concentration (mM) and kinetic constants were determined by fitting the data to the modified Hill equation, as described elsewhere (Ballicora et al., 2007), using the Levenberg–Marquardt non-linear least-squares algorithm provided by the computer program Origin^TM^ 8.0. These plots were used to calculate the Hill coefficient (*n*_H_) and the *S*_0.5_, defined as the substrate concentration giving 50% of the maximal velocity (*V*_max_). Kinetic constants are the mean of at least three sets of data, which were reproducible within ±10%.

### Molecular Mass Determination

To determine the native structure of the recombinant proteins, the purified enzymes were subjected to gel filtration chromatography. Samples were loaded in a Superdex 200 HR 10/30 column (GE-Healthcare) previously equilibrated with *buffer C* [25 mM Tris-HCl pH 8.0, 100 mM NaCl, and 0.1 mM EDTA]. The molecular mass was calculated using the calibration plot constructed with protein standards, including thyroglobulin (669 kDa), ferritin (440 kDa), aldolase (158 kDa), conalbumin (75 kDa), and ovalbumin (44 kDa). The column void volume was measured using a blue dextran solution (Promega).

To study the effect of oligomerization on GalU and GalF activity, the purified enzymes were diluted fivefold (final concentration 5 mg/ml) with 100 mM HEPES-NaOH pH 8.0 or 100 mM Tris-HCl pH 8.0 and incubated for 30 min at 4°C, as previously described (Kleczkowski et al., 2005). Following incubation, gel filtration chromatography was performed and 1 ml fractions were collected to determine kinetic parameters.

### Genetic Complementation of *galU^-^* Mutant

A strain carrying a mutation in *galU* gene, *E. coli* FF4001 (Harding et al., 1993), was transformed with pGALU, pGALF, pM15TH16R constructions, and the empty vector pMAB5. Transformed bacteria were plated in Hugh and Leifson (1953) base medium with 1% (w/v) D-Glc or 1% (w/v) D-Gal, to determine the capability of the cells to ferment the carbohydrate, as previously described (Hossain et al., 1994). Media was supplemented with 50 μg/ml kanamycin and 0.4 mM IPTG.

### Structure Prediction by Homology Modeling

The homology modeling was performed with the program Modeller 8v2 (Sali and Blundell, 1993). GalU model was constructed to include the product UDP-Glc and the Mg^2+^ ion from *Corynebacterium glutamicum* GalU (Protein Data Bank code: 2PA4). The crystal structure of *E. coli* GalU (Protein Data Bank code: 2E3D) and *C. glutamicum* GalU were used as a template for the GalF model. The model was checked with the programs Verify3D (Luthy et al., 1992). Only two regions of the GalF and GalU models fell below a score of 0.2, which is accepted as excellent. These were between residues 17–36 and 66–95 for GalF, and 18–37 and 67–95 for GalU. These lower scores were actually expected, since the homologous regions (18–39 and 67–86) in the GalU template had also a low score. There is an interaction between these regions in the quaternary structure to bridge two different subunits. Therefore, when the model of a monomer is analyzed, even for a structure solved by x-ray, side chains that are buried become exposed artificially lowering the score.

Figures were prepared with the program UCSF Chimera 1.11 (Regents of the University of California).

### Phylogenetic Analysis

Prokaryotic UDP-Glc PPases sequences from Supplementary Table S1 were downloaded from the NCBI database^1^ and classified into different groups using taxonomic data provided by the NCBI. A preliminary alignment was constructed using the ClustalW multiple sequence alignment server^2^ (Jeanmougin et al., 1998) and afterward, it was manually refined with the BioEdit 7.0 program^3^ (Hall, 1999). A rooted neighbor-joining tree based on the refined alignment was constructed using the accessory application in the SeaView 4.3 program^4^ (Gouy et al., 2010) using the Maximum likelihood method as implemented in the program. Finally, the tree was prepared with the FigTree 1.3 program^5^.

## Results

For a better understanding of the occurrence of the genes *galU* and *galF*, and the proteins encoded by them, we approached the study of the UDP-Glc PPase (GalU) and the homologous protein (GalF) found in *E. coli.* The genes *galU* (Gene ID: 945730) and *galF* (Gene ID: 946560) code for two proteins of 302 (GalU) and 297 amino acids (GalF), respectively. They share 57% of amino acid identity between each other and ∼30–45% identity with UDP-Glc PPases from other bacteria, such as *C. glutamicum* (Thoden and Holden, 2007b), *Helicobacter pylori* (Kim et al., 2010), *Sphingomonas elodea* (Aragao et al., 2007), *Streptococcus mutans* (Asencion Diez et al., 2013).

### Expression and Characterization of Recombinant GalU and GalF

**FIGURE 1:**
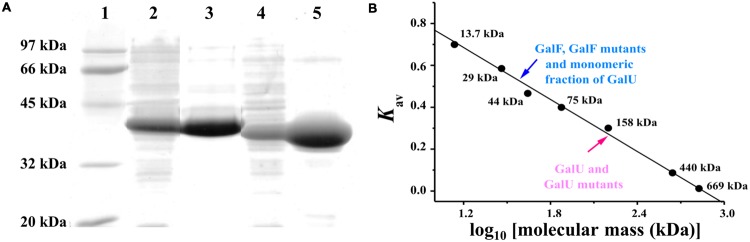
**Determination of GalU and GalF structures. (A)** Sodium dodecyl sulfate polyacrylamide gel electrophoresis (SDS-PAGE) of recombinant UDP-Glc PPases from *E. coli*. Lane 1: Molecular weight markers; Lane 2: GalU over-expression in crude extracts; Lane 3: Purified GalU; Lane 4: His-tagged GalF over-expression; Lane 5: Purified His-tagged GalF. **(B)** Molecular mass determination performed by size exclusion chromatography. Gel filtration chromatograms and molecular mass determination are shown in Supplementary Data.

We designed the primers to amplify the genes *galU* (909 bp) and *galF* (894 bp) from *E. coli* K-12 genomic DNA in a single-step PCR procedure as described in Experimental Procedures. After confirming identity by DNA sequencing, the amplified products were cloned into the commercial vector pET28c. *E. coli* BL21 (DE3) cells were transformed with those constructs to produce GalU and GalF, respectively. The last protein was expressed as a fusion to a His-tag, in order to avoid the co-purification of endogenous GalU from the host cell, so any possible activity measured is devoid of interferences. Both recombinant proteins were over-expressed in a soluble form (**Figure 1A**, lanes 2 and 4), and purified to high degree. They were more than 94% and 97% purity based on densitometry analysis for GalU and GalF, respectively, as described in Experimental Procedures (**Figure 1A**, lanes 3 and 5). To discard possible interferences from the His-tag, we also expressed GalF as an untagged protein. Results in characterization showed no significant structural or kinetic differences between the two versions of GalF (data not shown).

Both purified GalU and GalF proteins showed UDP-Glc PPase activity, but with marked differences in *V*_max_, being much higher for GalU (340 U/mg) than GalF (0.015 U/mg). As expected, both enzymes exhibited a strict dependence on Mg^2+^ to catalyze the synthesis of UDP-Glc and PP_i_ from UTP and Glc-1P. **Table 1** summarizes the assayed kinetic parameters for both enzymes. Both GalU and GalF exhibited saturation curves with a sigmoidal behavior for the essential cofactor, but GalF had slightly higher *S*_0.5_. In addition, GalF also had a higher *S*_0.5_ for UTP and a sigmoidal behavior, whereas GalU had a hyperbolic saturation curve. Besides the *V*_max_, the biggest difference was found in the parameters determined for Glc-1P: GalF exhibited a *S*_0.5_ one order of magnitude higher than GalU. In addition, GalF had a clear negative cooperativity for Glc-1P, whereas GalU only slightly deviated from a hyperbolic behavior.

**Table 1:** Kinetic parameters^a^ for GalU, GalF, and double mutants GalU_T20M/R21H_ and GalF_M15T/H16R_.

		GalU		GalU_T20M/R21H_		GalF_M15T/H16R_	GalF
		Tetramer	Monomer	Tetramer	Monomer	Monomer	Monomer
UTP	*S*_0.5_ (μM)	170 ± 20	140 ± 10	270 ± 40	250 ± 30	460 ± 40	360 ± 20
	*n*_H_	1.1 ± 0.1	1.4 ± 01	1.2 ± 0.1	1.2 ± 0.1	1.2 ± 0.1	1.4 ± 0.1
Glc-1P	*S*_0.5_ (μM)	35 ± 5	82 ± 3	2300 ± 200	2000 ± 100	750 ± 60	520 ± 60
	*n*_H_	1.2 ± 0.1	1.2 ± 0.1	1.7 ± 0.2	1.4 ± 0.2	0.95 ± 0.06	0.63 ± 0.05
Mg^2+^	*S*_0.5_ (μM)	2200 ± 100	2000 ± 100	2600 ± 100	2800 ± 300	2200 ± 100	3100 ± 0.1
	*n*_H_	3.7 ± 0.5	2.4 ± 0.3	2.8 ± 0.2	3.0 ± 0.2	3.3 ± 0.4	2.3 ± 0.2

**FIGURE 2:**
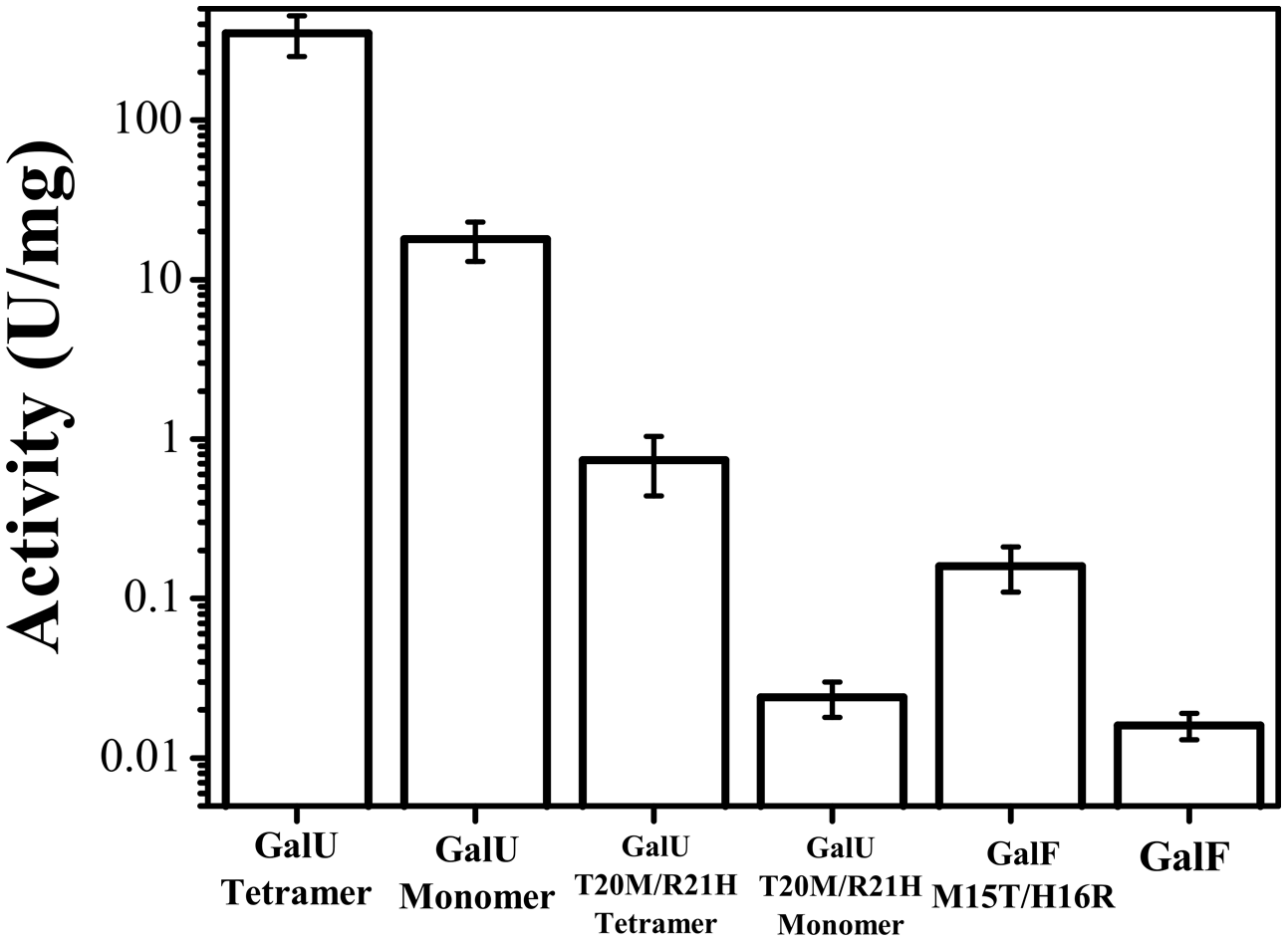
**Effect of the quaternary structure on the enzymes’ activities.** Activity was measured for the monomeric and tetrameric forms of GalU and its mutant GalU_T20M/R21H_ and compared to GalF and GalF_M15T/H16R_.

*^a^*V*_max_ values are shown in **Figure 2**.*

### Analysis of Quaternary Structure and Activity of the Enzymes

GalU and GalF quaternary structures were determined by size exclusion chromatography (**Figure 1B**). The former exhibited an elution profile of a homotetrameric protein (∼160 kDa, **Figure 1B**), which was in agreement with a previous structural analysis that revealed an arrangement of dimer of dimers in the crystallized protein (Thoden and Holden, 2007a). On the other hand, GalF ran as a monomer (∼40 kDa, **Figure 1B**). Based in these results, we decided to investigate whether the oligomerization status of GalU affects the activity of the enzyme; and to which degree the quaternary structure is responsible for the differences in the kinetic parameters.

It has been reported that the barley UDP-Glc PPase undergoes changes in oligomeric status by incubation in different buffers (Kleczkowski et al., 2005). Thus, GalU was incubated in HEPES-NaOH (pH 8.0) and Tris-HCl (pH 8.0) buffers and analyzed by size exclusion chromatography. After incubation in Tris-HCl, the enzyme eluted as a unique peak corresponding to a tetrameric form. Conversely, incubation in HEPES-NaOH promoted the de-oligomerization of the enzyme, which eluted as tetramers and monomers. The fractions were collected and kinetically analyzed. Interestingly, the monomeric GalU exhibited a *V*_max_ 20-fold lower (18 U/mg) than the tetrameric form (350 U/mg; **Figure 2**). However, this parameter was still higher (∼1000-fold) than the *V*_max_ exhibited by GalF. Regarding kinetic parameters for the substrates, the *S*_0.5_ for Glc-1P slightly increased (82 μM) compared to the value observed in the tetrameric form (35 μM). Parameters for UTP and Mg^2+^ remained nearly the same (**Table 1**). Similar analysis was performed with GalF, however, no difference in oligomerization status was observed for this protein.

### Analysis of Critical Residues in Pyrophosphorylase Homologs

There is a broad spectrum of similarity, which goes from 27 to 74% identity, among GalU and GalF proteins available in protein database. However, residues described as important to the activity of UDP-Glc PPases are conserved (Thoden and Holden, 2007b; Kim et al., 2010). However, GalF exhibited very low activity compared to GalU and other prokaryotic UDP-Glc PPases previously reported (Bonofiglio et al., 2005; Bosco et al., 2009; Asencion Diez et al., 2012, 2013). As described above, the quaternary structure adopted by these enzymes influences their activity. However, GalU even in its monomeric form had a higher activity than GalF. This led us to hypothesize that the absence of key residues may cause such kinetic discrepancies.

**FIGURE 3:**
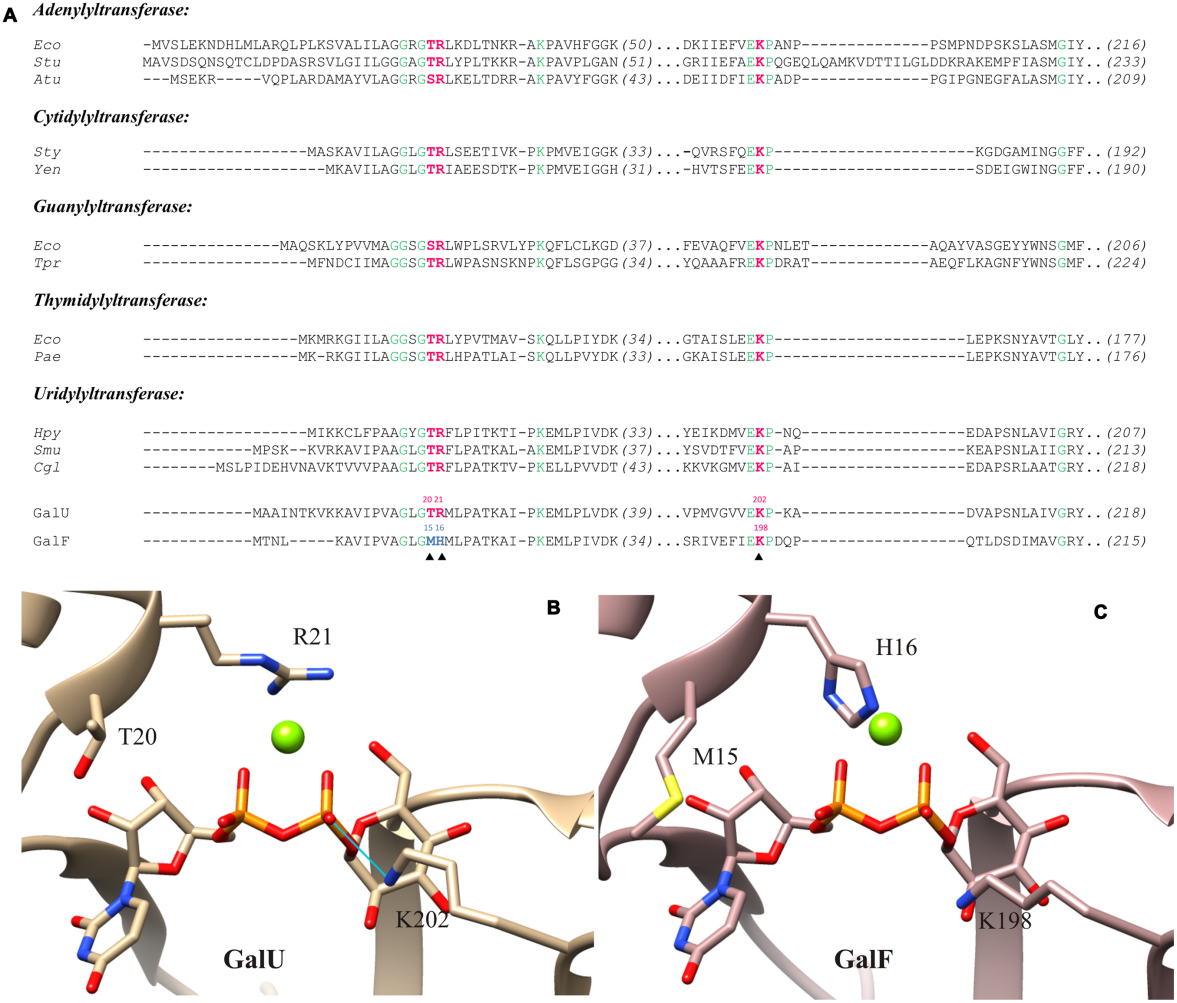
**Active site models of GalU and GalF. (A)** Sequence comparison of the UDP-Glc PPases (GalU and GalF) from *Escherichia coli* with pyrophosphorylases of different organisms. Residues 100% conserved are in green. Key residues analyzed in this study are in red. The key residues from GalF that are not conserved are shown in blue. Sequences and their accession numbers of adenylyltransferases (ADP-Glc PPase, EC: 2.7.7.27) are: Eco, *E. coli* K-12, P00584; Stu, *Solanum tuberosum* small subunit, P23509; Atu, *Agrobacterium tumefaciens*, P39669. Sequences and their accession numbers of cytidylyltransferases (α-D-glucose-1-phosphatecytidylyltransferase, EC: 2.7.7.33) are: Sty, *Salmonella typhi*, 1TZF; Yen, *Yersinia enterocolitica*, LC20, AHM71362. Sequences and their accession numbers of guanylyltransferases (GDP-mannose 1-phosphate guanylyltransferase, EC: 2.7.7.22) are: Eco, *E. coli* K-12, AAC75110; Tpr, *Treponema primitia*, WP_015708896. Sequences and their accession numbers of thymidylyltransferases (dTTP:α-D-glucose-1-phosphate thymidylyltransferase, EC: 2.7.7.24) are: Eco, *E. coli*, WP_000783975; Pae, *Pseudomonas aeruginosa*, WP_003105518. Sequences and their accession numbers of uridylyltransferases (UDP-Glc PPase, EC: 2.7.7.9) are: Hpy, *Helicobacter pylori*26695, 3JUJ_A; Smu, *Streptococcus mutans*, AGI47014; Cgl, *Corynebacterium glutamicum* ATCC 13032, NP_600109; GalU, *E. coli*, WP_000718996; GalF, *E. coli*, WP_001537247. **(B)** Structural model of GalU; the product, UDP-Glc, and the ion Mg^2+^ were inherited from *C. glutamicum* UDP-Glc PPase crystal structure (2PA4). Homology model shows key residues mutated in this study: T20, R21, and K202. The potential hydrogen bond between K202and the β-phosphate of the UDP-Glc is indicated by the blue line. **(C)** Homology model of GalF with the product and the cofactor added. Key residues are shown: M15, H16, and K198.

In order to identify those key functional residues, we analyzed the amino acid sequences and compared them with other pyrophosphorylases. It has been described that the motif GXG(T/S)R is highly conserved among NDP-sugar PPases (Jin et al., 2005) and it has been identified as part of the nucleotide binding site (Brown et al., 1999; Sivaraman et al., 2002; Jin et al., 2005; Koropatkin et al., 2005; Maruyama et al., 2007; Steiner et al., 2007; Pelissier et al., 2010). On the other hand, this motif is not conserved in the eukaryotic enzyme form *Leishmania major* (Führing et al., 2013). As shown in the sequence alignment of **Figure 3A**, this motif is present in GalU; but in GalF, residues T20 and R21 (GalU nomenclature) have been replaced by residues M15 and H16, respectively (GalF nomenclature).

We built a molecular model of GalF from *E. coli* (**Figure 3C**) with the positioning of the sugar-nucleotide and the essential divalent metal ion cofactor. For a proper comparison, those ligands were also modeled into the *E. coli* GalU (**Figure 3B**). As template for the three-dimensional modeling, *C. glutamicum* GalU was used, whose structure was solved with the product and Mg^2+^ (Protein Data Bank code: 2PA4; Thoden and Holden, 2007b). Thus, the UDP-Glc and Mg^2+^ were added from this crystal structure. The cation included in our models is the one bound by the α- and β-phosphoryl oxygen atoms of the UDP-Glc and the side chain of D142 in *C. glutamicum* GalU. This metal ion is also present in the thymidylyltransferase from *E. coli* at this position (Sivaraman et al., 2002), which is also near the Asp residue shown as essential for activity in ADP-Glc PPases (Frueauf et al., 2001). As shown in **Figure 3B**, the polar T20 and the charged R21 residues are part of the catalytic pocket in GalU. In GalF, the environment of the pocket is modified after replacement of these residues by M15and H16, respectively (**Figure 3C**).

In order to test whether these residues were important in the activity of the enzymes we constructed double-mutants of GalU (GalU_T20M/R21H_) and GalF (GalF_M15T/H16R_). Interestingly, the mutation in GalF_M15T/H16R_ increased the activity one order of magnitude (**Figure 2**) without significantly changing the *S*_0.5_of the substrates (**Table 1**). In addition, the hyperbolic kinetic behavior of GalF_M15T/H16R_ for Glc-1P made it more similar to GalU considering than GalF presents negative cooperativity (**Table 1**). However, the apparent affinity for Glc-1P has not been improved by the mutation. For the Mg^2+^ ion, the GalF_M15T/H16R_ mutation decreased *S*_0.5_ and increased the *n*_H_, to exhibit a kinetic behavior more similar to GalU (**Table 1**). Concurrently, the mutant GalU_T20M/R21H_had a much lower enzymatic activity than the wild type (*V*_max_ was three orders of magnitude lower, **Figure 2**). In addition, GalU_T20M/R21H_ increased 60-fold the *S*_0.5_ for Glc-1P (**Table 1**). The kinetic parameters for UTP and Mg^2+^ were similar for both the wild type and the double mutant forms of GalU (**Table 1**).

**FIGURE 4:**
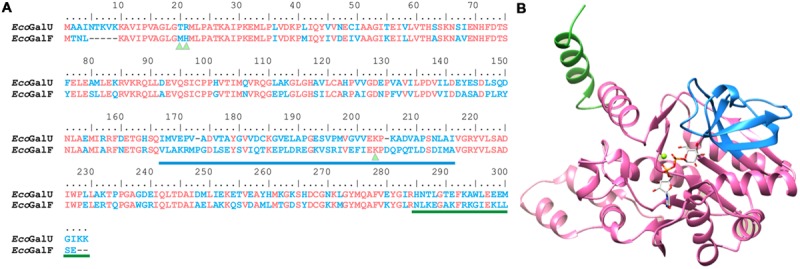
**Analysis of Glc-1P binding site. (A)** Sequence alignment of GalU and GalF from *E. coli*. Identical residues are shown in pink and difference in sequence in blue. Triangles indicate the residues analyzed in the present study. The loop involved in Glc-1P binding is underlined in blue and the dimerization fragment in green. **(B)** Homology model of *E. coli* GalU. Glc-1P binding site is shown in blue, and the divergent C terminus in green.

Another important structural region highly conserved among pyrophosphorylases (Hill et al., 1991; Thorson et al., 1994; Fu et al., 1998; Blankenfeldt et al., 2000; Koropatkin et al., 2005; Thoden and Holden, 2007b) comprises K202 in GalU (**Figure 3A**). The homology model (**Figure 3B**) shows that this residue interacts with the β-phosphoryl group of UDP-Glc, which implies it is important for the interaction with the phosphate of the Glc-1P. This indicates this lysine is responsible for a high affinity binding as it is in all the other sugar-nucleotide pyrophosphorylases studied so far. In GalF a homologous residue is present in the amino acid sequence (K198, see **Figures 3A** and **4**). However, analyzing the 3D model (**Figure 3C**), it seems that K198 is at a slightly longer distance to form a hydrogen bond with the UDP-Glc molecule.

All residues known to be involved in Glc-1P binding in the homologous *E. coli* ADP-Glc PPase (Bejar et al., 2006a) that are also present in GalU are indeed present in GalF (**Figure 4**). For instance, E201, K202, Y218, and D265 in GalU correspond to the residues E194, K195, Y216, and D276 in the *E. coli* ADP-Glc PPase, respectively. The corresponding homologous residues are E197, K198, Y215, and D262 in GalF. Most other contacts of the sugar phosphate seemed to be with the backbone of the polypeptide (Thoden and Holden, 2007a). For this reason, it is not obvious what residue may be missing to explain the low apparent affinity of GalF for Glc-1P. Another alternative is that there is a structural change that does not allow a proper interaction with the ligand. To test this hypothesis, we mutated K198 in GalF and K202 in GalU to see whether this residue is still important for Glc-1P binding as it is in all pyrophosphorylases known so far.

**FIGURE 5:**
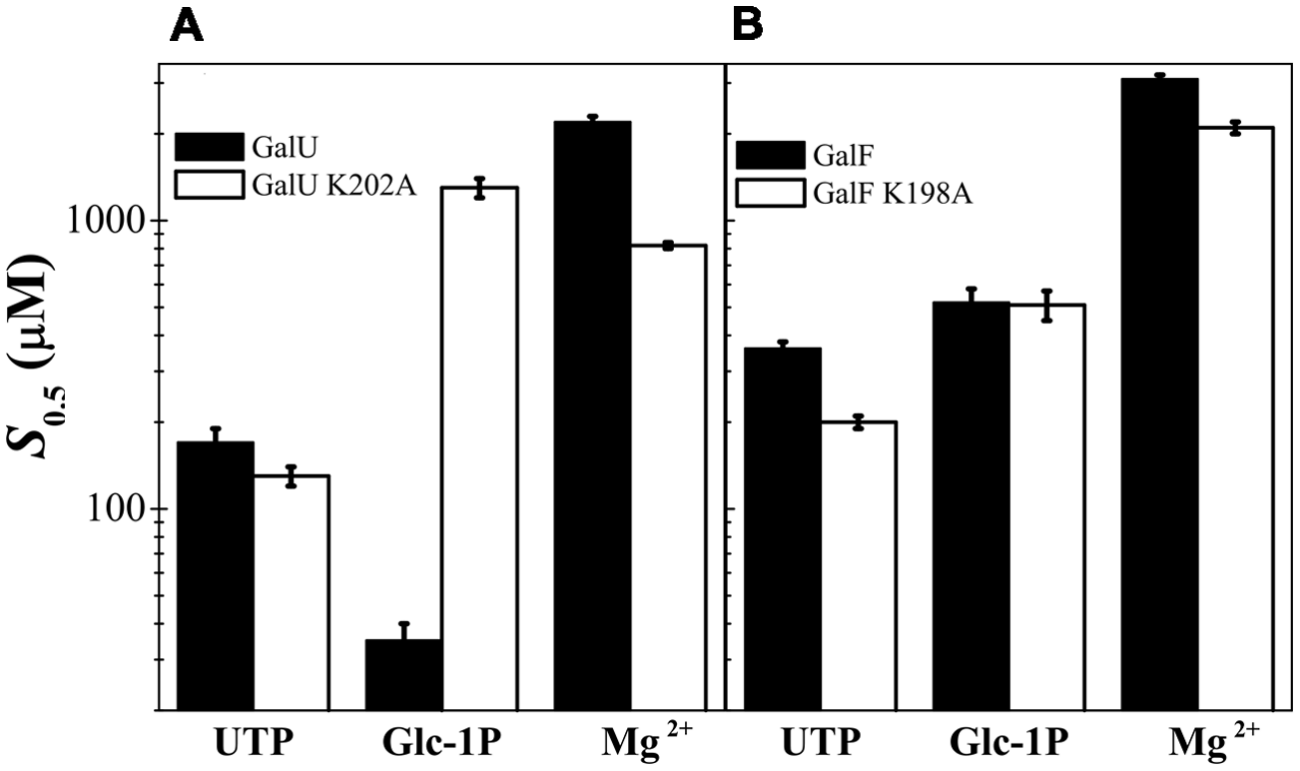
**Analysis of the substrates *S*_0.5_ for the mutations in Lys residue. (A)** Kinetic parameters determined for GalU and its mutant GalU_K202A_. **(B)** Parameters for GalF and GalF_K198A_.

We constructed site-directed mutants of GalU and GalF in the Lys residues (GalU_K202A_ and GalF_K198A_) and analyzed their kinetic properties. GalU_K202A_ exhibited a *V*_max_ similar to the wild type GalU; however, the *S*_0.5_ for the Glc-1P was dramatically increased by ∼40-fold (**Figure 5A**). Thus, the catalytic efficiency for the use of the substrate (given by the *V*_max_/*S*_0.5_ ratio, analogous to the catalytic efficiency *V*_max_/*K*_m_ in hyperbolic kinetics) decreased two orders of magnitude due to the absence of the K202 residue. For the UTP no significant changes in the kinetic parameters were observed. For the Mg^2+^ ion, the mutant GalU_K202A_ exhibited a *S*_0.5_ only ∼threefold lower than GalU (**Figure 5A**). These results indicated that K202 is involved in the binding of Glc-1P and it is consistent with previous data from other homologous NDP-Glc PPases (Hill et al., 1991; Fu et al., 1998) showed a *S*_0.5_ slightly lower than the wild-type enzyme (**Figure 5B**). In other words, unlike in GalU, the mutation K198A in GalF did not further alter the kinetic parameters for Glc-1P.

In all these mutagenesis experiments, the changes in primary structure did not produce a different quaternary structure: GalU_T20M/R21H_ and GalU_K202A_ exhibited a tetrameric conformation; and GalF_M15T/H16R_ and GalF_K198A_ behaved as monomers. Based on these results we hypothesized that the higher activity of GalU_T20M/R21H_ compared to GalF could be due to the difference in quaternary structure. Thus, we obtained the monomeric form of GalU_T20M/R21H_, as described above for GalU. The monomeric fraction did not show changes in apparent affinity of the effectors (**Table 1**), although the specific activity decreased a bit more than one order of magnitude, reaching a value similar to GalF (**Figure 2**).

### Complementation of the *E. coli* Mutant Strain *galU*^-^

In this work we demonstrated that GalF has a residual UDP-Glc PPase activity. Considering only these results it might be possible that this enzyme could compensate the absence of GalU. However, it is known that *E. coli* null mutants in *galU* gene cannot ferment Gal and fail to incorporate Glc and Gal into bacterial cell membranes yielding an incomplete synthesis of LPS (Weissborn et al., 1994). One possible explanation to this physiological characteristic is that the *in vivo* production of UDP-Glc from GalF is not sufficient for the cellular metabolism. From this assumption, an increase in the expression of this enzyme could overcome the limitation of the low enzymatic activity. In addition, the expression of GalF_M15T/H16R_, which is more active than GalF, might be more efficient to complement a *galU^-^* mutant.

**FIGURE 6:**
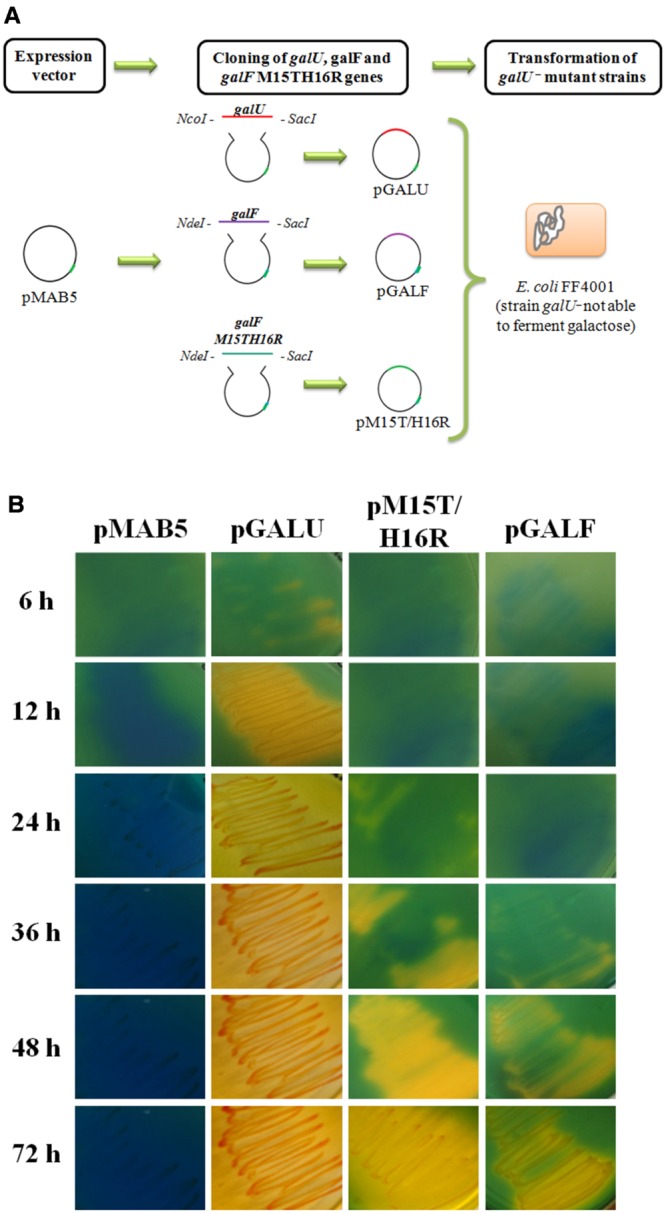
**Complementation assays of *E. coli* strain *galU*^-^. (A)** Schematic representation of the strategy used for the assay. **(B)** *E. coli* strain *galU*^-^ transformed with pMAB5 (control) and constructions pGALU, pGALF, and pM15T/H16R. Cultures were grown at 37°C in the Hugh–Leifson medium with 1% (w/v) Gal. Yellow indicates fermentation of the sugar and blue indicates inability to use Gal as a carbon source.

To probe the possible *in vivo* functionality of GalF and GalF_M15T/H16R_, we transformed *E. coli* FF4001 [an *E. coli* strain carrying a mutation in *galU* (Harding et al., 1993)] with plasmids pGALU, pGALF, pM15T/H16R, and the vector pMAB5 (**Figure 6A**). This *E. coli* mutant has altered the Leloir (1971) pathway and is unable to grow using Gal as a carbon source. Cells carrying the plasmids were grown in Hugh and Leifson (1953) medium supplemented with Gal or Glc (as control, data not shown) as described in “Materials and Methods” section. Color change of the culture medium to yellow indicates acidification due to sugar consumption; whereas color blue indicates the inability of the culture to ferment Gal. **Figure 6B** shows cells transformed with plasmids pGALU, pGALF, pM15TH16R, and pMAB5 after different incubation times at 37°C. The expression of the enzymes allowed the bacteria to consume the Gal. In good agreement with the *in vitro* results (**Table 1**), cells transformed with pGALU metabolized Gal within the first 12 h of incubation, whereas those complemented with pM15T/H16R and pGALF required a longer time to consume of the sugar (48 and 72 h, respectively). During the whole course of the control experiment, cells were unable to use Gal as a carbon source (**Figure 6B**).

## Discussion

In the present study, we kinetically and biochemically characterized the product of two genes from *E. coli* that encode the prokaryotic UDP-Glc PPase (GalU) and a putative homologous enzyme (GalF). The genes were amplified by PCR and cloned into an expression vector to produce the recombinant proteins. Both purified enzymes had the ability to catalyze the reaction in certain conditions; although they exhibited dramatically different kinetic and structural properties. The most outstanding difference between them was the *V*_max_. This value for GalF was four orders of magnitude lower than the observed for GalU. This should clarify some contradictory statements in the literature. This *E. coli* GalF protein was reported as inactive, but those assays were performed on crude extracts (Marolda and Valvano, 1996). Previously, several groups (Schnaitman and Klena, 1993; Varon et al., 1993; Hossain et al., 1994; Macpherson et al., 1994; Thorson et al., 1994) postulated that GalU and GalF were isozymes of UDP-Glc PPase on the basis of amino acid sequence similarity. Also, it has been proposed that *E. coli* GalF is a regulatory subunit of GalU without functional similarity (Marolda and Valvano, 1996). Conversely, it has been suggested that in *Klebsiella pneumoniae* GalF could work as virulence factor (Ho et al., 2011). The main reason was that the production of UDP-Glc was critical for the biosynthesis of the capsular polysaccharide (K antigen) and the authors assumed GalF was responsible for it. However, the bulk of catalysis for the production of UDP-Glc *in vivo* most likely did not come from GalF. Some UDP-Glc PPase activity was qualitatively detected for GalF after incubating a reaction mixture with 10 μg of recombinant protein for 4 h (Ho et al., 2011). They did not report the specific activity of *K. pneumoniae* GalF, but considering the long incubations and high concentrations of enzyme used (Ho et al., 2011); the data provided is compatible with the values we report in this paper. A specific activity of *E. coli* GalF (0.015 U/mg or turnover number of 0.01 s^-1^) is negligible compared to the specific activity of GalU (340 U/mg or 227 s^-1^). Also, we observed that GalF is not enough to complement the absence of GalU to ferment Gal at similar rates. Most likely, the synthesis of UDP-Glc in *Klebsiella* comes from GalU (Accession # BAH63931). A low activity of GalF in *Klebsiella* is compatible with the fact that the motif GXG(T/S)R is disrupted as in the *E. coli* GalF, since it has a Met and His rather than Thr and Arg (Accession # KP3726).

Considering that GalU and GalF are homologous proteins (57% of identity) it was necessary to analyze what changes in protein structure could explain the differences. We hypothesized that this could be due to: (i) the absence of key residues in GalF and/or (ii) the occurrence of residues affecting its interaction with the substrates and (iii) the difference in its quaternary structure. *In silico* analysis allowed us to identify two conserved residues among all the pyrophosphorylases that are clearly part of the active site in GalU; but absent in GalF. Based on this, we constructed double mutants GalU_T20M/R21H_ and GalF_M15T/H16R_ to test the above hypothesis and single mutants GalU_K202A_ and GalF_K198A_to probe the active site. Results supported the relevance of the motif GLGTR (residues 17–21) in GalU. Mutations in GalU of this motif decreased the specific activity ∼500-fold, whereas the double mutant of GalF partially resurrected the enzyme activity by 10-fold. In good agreement with the above results, the Arg residue from different NDP-sugar PPases homologous to R21 in GalU has been postulated to be important for the enzyme catalysis (Brown et al., 1999; Sivaraman et al., 2002; Jin et al., 2005; Koropatkin et al., 2005; Maruyama et al., 2007; Steiner et al., 2007; Pelissier et al., 2010). The GXG(T/S)R loop is thought to bind the β- and γ-phosphates of the NTP (Jin et al., 2005). The Arg would be one of the residues responsible to counterbalancing negative charges of the phosphate moieties, leading to the correct position of NTP to further facilitate the binding of Glc-1P (Blankenfeldt et al., 2000; Sivaraman et al., 2002; Jin et al., 2005). It has been noticed that UDP-Glc PPase is structurally similar to Glc-1P thymidylyltransferase (Blankenfeldt et al., 2000) and UDP-*N*-acetylglucosamine PPase (GlmU; Brown et al., 1999). In addition, there is a lower but still significant structural identity of GalU to ADP-Glc PPase (Bejar et al., 2006b) and the Glc-1P cytidylyltransferase (Thorson et al., 1994). Previous works showed that mutation of homologous Arg in different PPases significantly affected the activity. For the potato tuber ADP-Glc PPase, we reported a reduction in the activity when R33 of the catalytic small subunit was mutated (Ballicora et al., 2005). Also, the enzymatic activity of its modulatory large subunit was resurrected by mutating the homologous residue K44 to Arg. For the *Agrobacterium tumefaciens* ADP-Glc PPase the study of N-terminal Arg residues showed the importance of R25 (homologous to R21 in GalU) for the catalysis (Gomez Casati et al., 2001). The change of this residue to Ala decreased the *V*_max_ in two orders of magnitude and increased the ATP *S*_0.5_ in one order of magnitude. In a same way, mutations in the homologous Arg in the *Helicobacter pylori* UDP-Glc PPase (R15A mutant; Kim et al., 2010) and in the *E. coli* GlmU (R18A mutant; Brown et al., 1999) reduced the activity 86% and 6000-fold, respectively. On the other hand, not all UDP-Glc PPases have this important Arg. It seems it is a characteristic of prokaryotic UDP-Glc PPases, since the eukaryotic enzyme from *Leishmania major* does not have it, and the binding of the gamma phosphate is facilitated by a different array of interactions (Führing et al., 2013).

It is evident that the substitution of the Arg/Thr residues in GalF is not the only reason for the low activity. Another possible factor could be the occurrence of residues that affect the correct binding of the substrates. The homology model of GalU showed that K202 interacts with the β-phosphoryl group of UDP-Glc, as it was observed for *C. glutamicum* GalU, Glc-1P thymidylyltransferase from *Pseudomonas aeruginosa*, and Glc-1P cytidylyltransferase from *Salmonella typhi* (Thoden and Holden, 2007b). In accordance to this, the mutant GalU_K202A_ markedly increased the Glc-1P *S*_0.5_. The Lys residue is highly conserved in the family. Pioneer work in the *E. coli* ADP-Glc PPase (Hill et al., 1991) showed an important role of the homologous K195 for the proper binding of Glc-1P at the catalytic site. In GalF the Lys residue is present (K198), but the surrounding sequence has been heavily modified. In GalF, the subdomain responsible for the Glc-1P binding has a remarkable low identity to the homologous one in GalU (I167 to I214). This region shares an identity of only 21% whereas the rest is 63% (the overall identity is 57%). The kinetic characterization was in agreement with this sequence analysis. GalF exhibited a greater *S*_0.5_ for Glc-1P (∼15-fold higher) than GalU and the mutant GalF_K198A_ was not significantly different from the wild-type GalF. This structural characteristic suggests that the proper substrate binding site is already altered in GalF, in such a way that the K198 already exerts a diminished effect. This also explains the reduced apparent affinity of GalF for Glc-1P when compared with GalU. This view agrees with a combined analysis of the results obtained with double mutants GalU_T20M/R21H_ and GalF_M15T/H16R_, where the amino acid replacement affects the apparent affinity for Glc-1P in GalU but not in GalF. This suggests that in the latter such structural modification is minimized because the substrate binding site is already disrupted. The presence of Met and His rather than Thr and Arg causes a decrease of apparent affinity for Glc-1P in GalU. Based on the crystal structure of GalU (Thoden and Holden, 2007a,b), T20 and R21 would be near the nucleotide UTP rather than Glc-1P, but it is possible that alteration of the first substrate positioning (UTP) could modify the apparent affinity for the second (Glc-1P). It is already known that in this enzyme family, the nucleotide binds first (Zuccotti et al., 2001).

**FIGURE 7:**
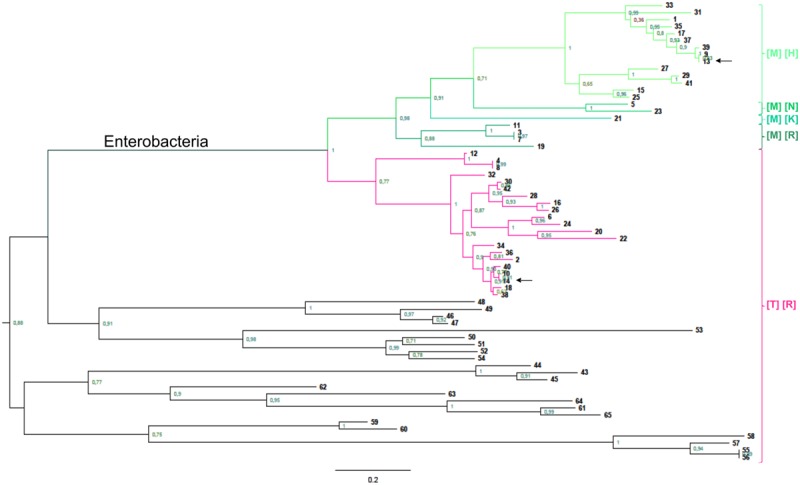
**Phylogenetic analysis of GalU and GalF.** Phylogenetic relationship of prokaryotic UDP-Glc PPases. The rooted trees were built as described in Experimental Procedures, and reference numbers in bold are listed on Supplementary Table S2. The scale on the bottom left represents the number of substitutions per site. Green branches belong to GalF proteins, whereas pink branches are GalU proteins from enterobacteria. Between brackets, it is indicated the residues conserved in each group in the positions corresponding to T20 (first bracket) and R21 (second bracket) in *E. coli* GalU. Arrows indicate the sequences of *E. coli* proteins.

Phylogenetic analysis of the GalF protein shows that it evolved from a GalU in enteric bacteria. However, GalF branches are clearly longer implying a faster evolution (**Figure 7**). A closer inspection of the alignment between GalF and GalU identifies a distinct fraction of the protein. This fragment is in fact, as mentioned above, the domain responsible for Glc1P binding in nucleotidyl transferases. Building separate trees, for this Glc-1P domain (Supplementary Figure S1) and the rest of the protein (Supplementary Figure S2), shows the Glc-1P domain is the sole responsible for the higher divergence. The GalF and GalU branches have similar length when the Glc1P domain is absent (Supplementary Figure S2). If only the Glc1P domain is analyzed we will observe the opposite (Supplementary Figure S1). One possibility is that this domain was exchanged by recombination with other nucleotidyl transferase gene, but it does not seem to be the case because its similarity to other enzymes of the superfamily is even lower than with GalU. The alternative is that this domain evolved more rapidly because the protein acquired a new non-catalytic role, and as a consequence the constraints for each of the domains changed.

GalU and the mutants analyzed in the present work are homotetramers [as previously reported (Thoden and Holden, 2007a)], whereas GalF and its mutants are expressed as monomers. A number of enzymes in the family have both active oligomeric and inactive monomeric forms (or vice versa; Kleczkowski et al., 2005). In fact, in several cases (Torshin, 1999; Peneff et al., 2001; Wilczynska et al., 2003) oligomerization is one of the key regulatory processes that affect function/activity. Thus, the difference in oligomerization status could be a relevant factor affecting the activity of GalF. In fact, the monomeric fraction of GalU exhibited lower activity (20-fold) than the tetrameric form. The monomeric form of the mutant GalU_T20M/R21H_ exhibited a similar activity to GalF. Structural analysis of *E. coli* GalU revealed that the protein is a tetramer organized as a dimer of dimers (Thoden and Holden, 2007a). The C-terminus of this protein presents two helices (K269–R282 and G287–M298) that form the “tight” dimer by subunit–subunit interaction (Thoden and Holden, 2007a). Analysis of the amino acid sequences shows that GalF C-terminus significantly differs from GalU (**Figure 4**). Most likely, this difference is responsible for the monomeric form of GalF and its mutants.

The product of the gene *galU* is essential for Gal metabolism. Bacteria not expressing GalU cannot ferment this sugar to use it as a carbon source. This physiological effect can be reverted with the over-expression of GalF and GalF_M15T/H16R_. Complementation assays validated our findings: GalF in high concentrations is active as a UDP-Glc PPase. Endogenous GalF cannot produce enough amount of UDP-Glc as GalU does, but this limitation can be overcome in the laboratory by increasing the expression of the enzyme. Furthermore, expression of the double mutant form with a partially resurrected activity (GalF_M15T/H16R_) was more successful in allowing a *galU^-^* strain of *E. coli* to ferment Gal *in vivo*. There is an important conclusion from these experiments. It is risky to assign physiological roles based on overexpression of proteins without assaying specific kinetic constants of a particular enzyme. Non-physiological high concentrations of an enzyme may hide the fact that a much lower *k*_cat_ may impedes a functional catalytic role *in vivo*. In addition, since there is no expression of GalU in this particular strain with no possibility of endogenous activity or that a GalU subunit could be part of the quaternary structure of GalF, the results indicate that GalF has a residual enzyme activity of its own. These *in vivo* experiments support what we observed in the *in vitro* experiments when we overexpressed GalF and purified it with a HisTag.

As a whole, GalU and GalF are homologous proteins that share a different degree of catalytic activity. However, GalF showed structural modifications which led to dramatic kinetic differences from GalU. Similar cases between other pair of proteins have been described in which the non-enzyme proteins were formed from enzymes homologs, many times carrying substitutions at the catalytic sites (Todd et al., 2002; Pils and Schultz, 2004). This is a process that sometimes could be reverted *in vitro* by the mutation of only one or two residues (Ballicora et al., 2005). Many enzymes become non-enzymes to evolve new regulatory roles (Thornton, 2004; Ballicora et al., 2005). Thus, these processes have involved gene duplication, divergence, and functional evolution from a common ancestor. The low activity of GalF suggests that divergence from a common ancestor and evolutionary adaptations led to two proteins with different roles, and only one of them (GalU) kept the catalytic physiological function. Presumably, *galF* is a duplication of the gene *galU* and subsequent mutations produced the loss of activity. This natural mechanism, which might be a subfunctionalization process (Kuhn et al., 2009), could be a strategy for acquiring regulatory properties in GalF or a consequence of it.

## Author Contributions

Conceived and designed the experiments: AI and MB. Performed the experiments: AE, NS, and AO. Analyzed the data: AE, CF, AI, and MB. Contributed reagents/materials/analysis tools: AI MB. Wrote the paper: AE, CF, AI, and MB.

## Conflict of Interest Statement

The authors declare that the research was conducted in the absence of any commercial or financial relationships that could be construed as a potential conflict of interest.

## Footnotes

1 http://www.ncbi.nlm.nih.gov/

2 http://www.genome.jp/tools/clustalw/

3 http://www.mbio.ncsu.edu/bioedit/bioedit.html

4 http://pbil.univ-lyon1.fr/software/seaview.html

5 http://tree.bio.ed.ac.uk/
