# Supplementary material for: On the Ancestral UDP-Glucose Pyrophosphorylase Activity of GalF from Escherichia coli
Source: Front Microbiol. 2015 Nov 13;6:1253. doi: 10.3389/fmicb.2015.01253 (PMC4643126; doi:10.3389/fmicb.2015.01253)
Supplement: Supplementary file 1 [file Data_Sheet_1.DOCX]

Supplementary Material

Article Title

Ana C. Ebrecht; Agnieszka M. Orlo; Natalia Sasoni; Carlos M. Figueroa;
Alberto A. Iglesias; Miguel A. Ballicora^*^

*** Correspondence:** Corresponding Author: [mballic@luc.edu](mailto:mballic@luc.edu)

**1. Supplementary data.**

**1.1. Molecular Mass determination:**

**K_av_ = V_o_ * (V_t_ – V_o_) -V_e_**

Where:

V_e_= elution volume is measured from the chromatogram and relates to the molecular size of the molecule.

V_o_= voide volume is the elution volume of molecules that are excluded from the gel filtration medium because they are larger than the largest pores in the matrix and pass straight through the packed bed.

V_t_= total column volume is equivalent to the volume of the packed bed.

**
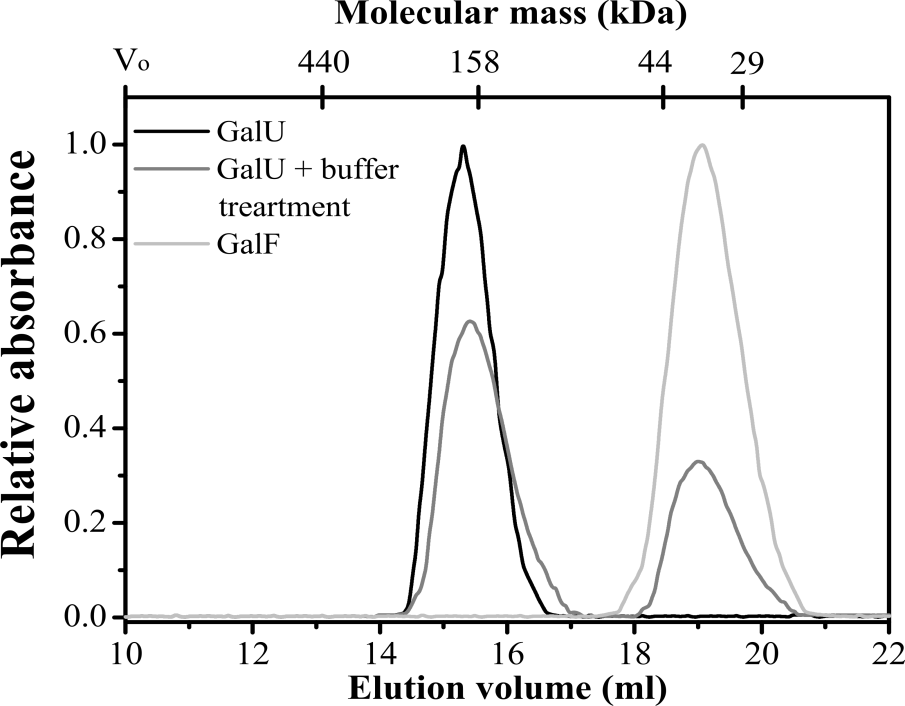
1.2. Gel filtration of GalU and GalF.** Enzymes were loaded into Superdex 200 column as detailed in Materials and Methods. Size exclusion chromatography was performed to GalU (**―**) and GalF (**―**) after purification. And tetrameric and monomeric GalU after treatment with buffer Hepes (**―**) is shown.

**2.2. Supplementary Figures and Tables**

**2.1. Supplemental Figure 1.** Phylogenetic analysis of Glc-1P binding site. Rooted tree was built using Glc-1P binding site (I167 to I214 from *E. coli* GalU) sequences of GalU and GalF of different bacteria.
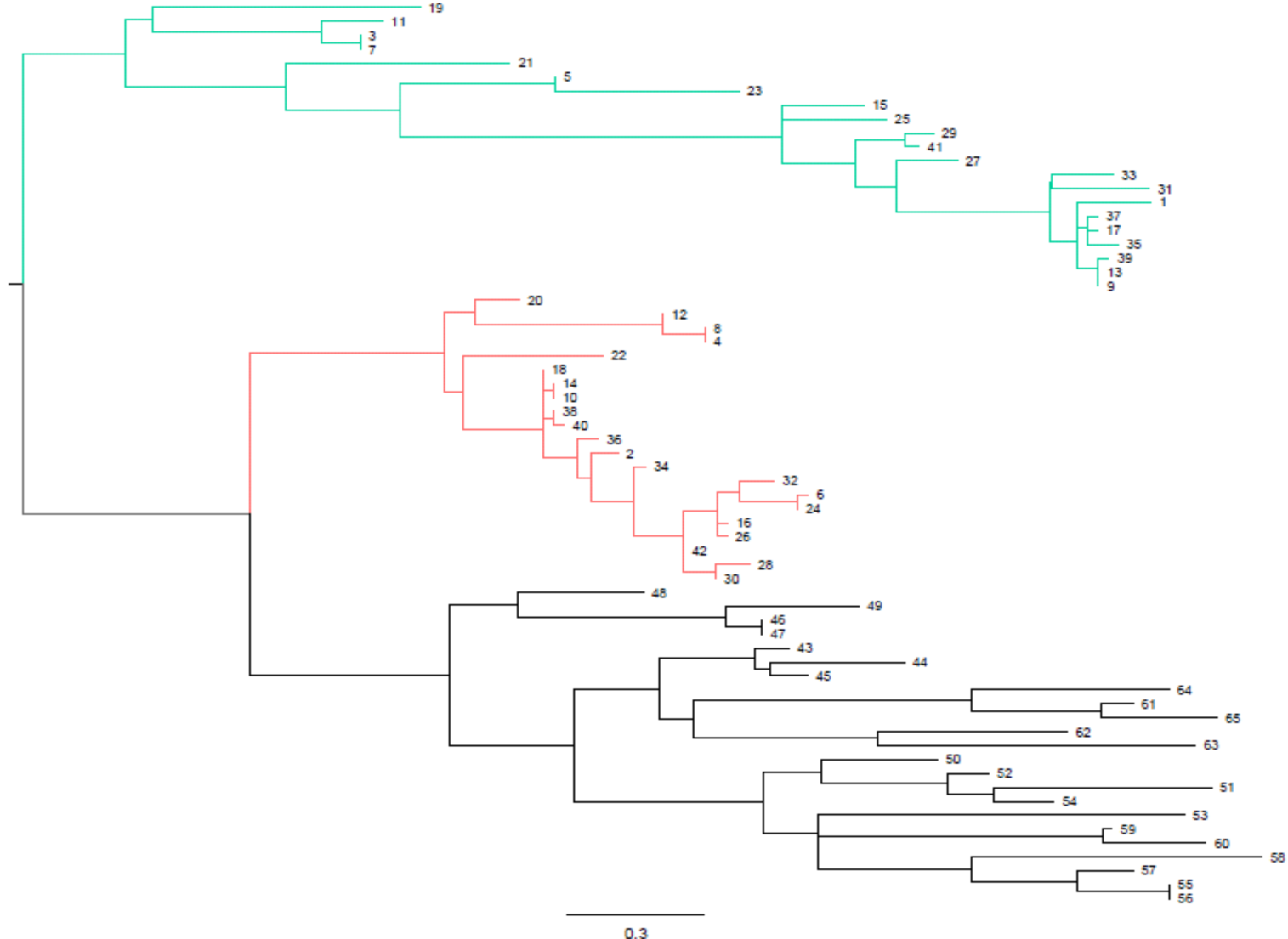


**2.2. Supplemental Figure 2.** Phylogenetic tree of GalU and GalF sequences without the Glc-1P binding site.
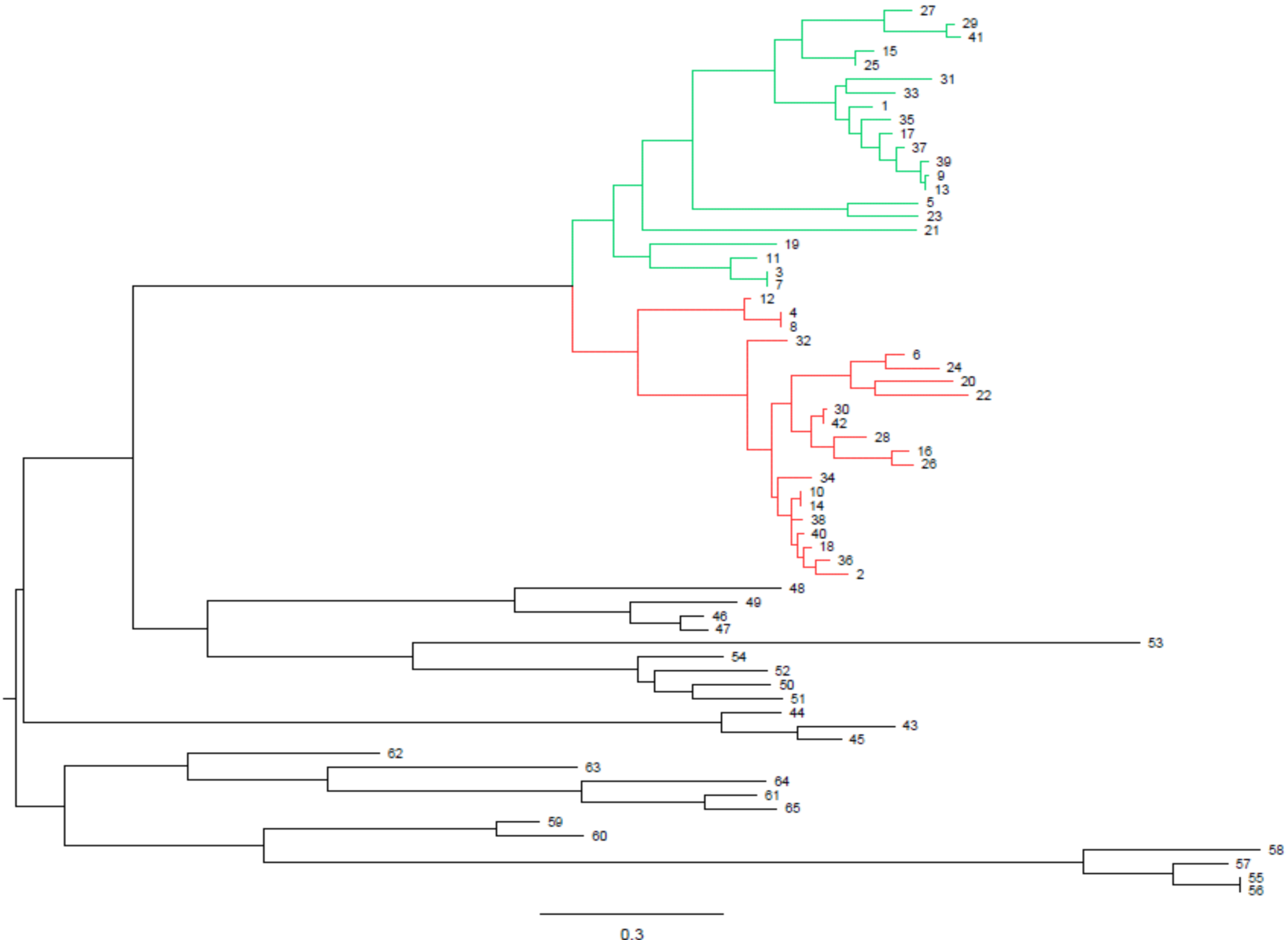


**2.3. Supplemental Table 1. Oligonucleotide sequences of the specific primers for *galU* and *galF* genes amplification (restriction sites are underlined) and for site-directed mutagenesis (changed bases are in bold).** Restriction sites for *galU*: *Nco*I (forward primer) and *Sac*I (reverse primer). Restriction sites for *galF*: *Nde*I (forward primer) and *Sac*I (reverse primer).

| **Primer** | **Oligonuclotide sequence** |
| --- | --- |
| *galU*WT-fo  *galU*WT-re | 5`˗CCATGGATGGCTGCCATTAATACGAAAGTCAAAAAAGCCGTTATCCCCGTTGCGGG-3`  5`˗GAGCTCTTATTCGCTTAACAGCTTCTCAATACCTTTAAATTCCGTGC-3` |
| *galU*T20MR21H-fo  *galU*T20MR21H-re | 5`- GCGGGATTAGGA**ATGCAT**ATGTTGCCGGCG-3`  5`- CGCCGGCAACAT**ATGCAT**TCCTAATCCCGC-3` |
| *galU*K202A-fo  *galU*K202A-re | 5`-GTGGTAGAA**GCG**CCGAAAGCG-3`  5`-CGCTTTCGGG**CGC**TTCTACCAC-3` |
| *galF*WT-fo  *galF*WT-re | 5`˗CATATGACGAATTTAAAAGCAGTTATTCCTGTAGCGGGTCTCGGGATGCATAT-3`  5`˗GAGCTCTTATTCGCTTAACAGCTTCTCAATACCTTTACGGAACTTCGCCCCTTCTT-3` |
| *galF*M15TH16R-fo  *galF*M15TH16R-re | 5`-GGTCTCGGG**ACCCGT**ATGTTGCCT-3`  5`-AGGCAACAT**ACGGGT**CCCGAGACC-3` |
| *galF*K198A-fo  *galF*K198A-re | 5`- GAATTTATCGAA**GCG**CCGGATCAGCCG-3`  5`- CGGCTGATCCG**GCG**CTTCGATAAATTC-3` |

**2.4. Supplemental Table 2.** Data of sequences used for the phylogenetic tree. The table contains the number used for each sequence, the corresponding NCBI accession number and annotation, the name of the organism, and the taxonomic group.

| **No.** | **Accenssion** | **Annotation** | **Organism** | **Taxonomy** |
| --- | --- | --- | --- | --- |
| **1** | BAH64302 | UTP-glucose-1-phosphate uridylyltransferase. Gene *galF* | *Klebsiella pneumoniae* subsp. *pneumoniae* NTUH-K2044 | Enterobacteria |
| **2** | BAH63931 | glucose-1-phosphate uridylyltransferase. Gene *galU* | *Klebsiella pneumoniae* subsp. *pneumoniae* NTUH-K2044 | Enterobacteria |
| **3** | AAM86184 | probably a UDP-gal transferase | *Yersinia pestis* KIM10+ | Enterobacteria |
| **4** | AAM86185 | glucose-1-phosphate uridylyltransferase | *Yersinia pestis* KIM10+ | Enterobacteria |
| **5** | CAG74353 | UTP--glucose-1-phosphate uridylyltransferase. Gene *galF* | *Pectobacterium atrosepticum* SCRI1043 | Enterobacteria |
| **6** | CAG75232 | UTP--glucose-1-phosphate uridylyltransferase. Gene *galU* | *Pectobacterium atrosepticum* SCRI1043 | Enterobacteria |
| **7** | CAH20791 | UTP-glucose-1-phosphate uridylyltransferase. Gene *galF* | *Yersinia pseudotuberculosis* IP 32953 | Enterobacteria |
| **8** | CAH20790 | UTP-glucose-1-phosphate uridylyltransferase. Gene *galU* | *Yersinia pseudotuberculosis* IP 32953 | Enterobacteria |
| **9** | YP_403779 | UTP-glucose-1-phosphate uridylyltransferase. Gene *galF* | *Shigella dysenteriae* Sd197 | Enterobacteria |
| **10** | ABB61435 | UTP-glucose-1-phosphate uridylyltransferase. Gene *galU* | *Shigella dysenteriae* Sd197 | Enterobacteria |
| **11** | CAL12810 | UTP-glucose-1-phosphate uridylyltransferase. Gene *galF* | *Yersinia enterocolitica* subsp. *enterocolitica* 8081 | Enterobacteria |
| **12** | CAL12811 | UTP-glucose-1-phosphate uridylyltransferase. Gene *galU* | *Yersinia enterocolitica* subsp. *enterocolitica* 8081 | Enterobacteria |
| **13** | AIZ51849 | putative regulatory subunit for GalU | *Escherichia coli* K-12 | Enterobacteria |
| **14** | AIZ51035 | glucose-1-phosphate uridylyltransferase | *Escherichia coli* K-12 | Enterobacteria |
| **15** | ADO10155 | UTP-glucose-1-phosphate uridylyltransferase. Gene *galF* | *Pantoea vagans*C9-1 | Enterobacteria |
| **16** | ADO09727 | glucose-1-phosphate uridylyltransferase. *galU* | *Pantoea vagans*C9-1 | Enterobacteria |
| **17** | NP_456647 | UTP-glucose-1-phosphate uridylyltransferase. Gene *galF* | *Salmonella enterica* subsp. *enterica serovar Typhi* str. CT18 | Enterobacteria |
| **18** | NP_455748 | glucose-1-phosphate uridylyltransferase. Gene *galU* | *Salmonella enterica* subsp. *enterica serovar Typhi* str. CT18 | Enterobacteria |
| **19** | AGE17370 | putative regulatory subunit for GalU | *Serratia marcescens* WW4 | Enterobacteria |
| **20** | AGE18517 | glucose-1-phosphate uridylyltransferase | *Serratia marcescens* WW4 | Enterobacteria |
| **21** | WP_005284178 | UTP--glucose-1-phosphate uridylyltransferase subunit GalU | *Edwardsiella tarda* | Enterobacteria |
| **22** | WP_005285150 | UTP--glucose-1-phosphate uridylyltransferase subunit GalU | *Edwardsiella tarda* | Enterobacteria |
| **23** | ADM97625 | predicted regulatory subunit of UTP--glucose-1-phosphate uridylyltransferase, with GalU | *Dickeya dadantii* 3937 | Enterobacteria |
| **24** | ADM98404 | UTP--glucose-1-phosphate uridylyltransferase | *Dickeya dadantii* 3937 | Enterobacteria |
| **25** | CCF08984 | UTP-glucose-1-phosphate uridylyltransferase GalF | *Pantoea ananatis*LMG 5342 | Enterobacteria |
| **26** | CCF09469 | UTP-glucose-1-phosphate uridylyltransferase GalU | *Pantoea ananatis* LMG 5342 | Enterobacteria |
| **27** | CAX60461 | UTP-glucose-1-phosphate uridylyltransferase. Gene *galF* | *Erwinia billingiae* Eb661 | Enterobacteria |
| **28** | CAX59937 | UTP-glucose-1-phosphate uridylyltransferase. Gene *galU* | *Erwinia billingiae* Eb661 | Enterobacteria |
| **29** | CAY73871 | UTP-glucose-1-phosphate uridylyltransferase. Gene *galF* | *Erwinia pyrifoliae* DSM 12163 | Enterobacteria |
| **30** | CAY74149 | glucose-1-phosphate uridylyltransferase. Gene *galU* | *Erwinia pyrifoliae* DSM 12163 | Enterobacteria |
| **31** | AFJ46548 | putative UTP-glucose-1-phosphate uridylyltransferase | *Shimwellia blattae* DSM 4481 = NBRC 105725 | Enterobacteria |
| **32** | AFJ46987 | UTP-glucose-1-phosphate uridylyltransferase | *Shimwellia blattae* DSM 4481 = NBRC 105725 | Enterobacteria |
| **33** | AFJ98988 | UTP--glucose-1-phosphate uridylyltransferase subunit GalF | *Cronobacter sakazakii* ES15 | Enterobacteria |
| **34** | AFJ99333 | UTP--glucose-1-phosphate uridylyltransferase subunit GalU | *Cronobacter sakazakii* ES15 | Enterobacteria |
| **35** | AIV30576 | UTP--glucose-1-phosphate uridylyltransferase subunit GalU. regulatory protein GalF | *Enterobacter cloacae* | Enterobacteria |
| **36** | KGB12686 | UTP--glucose-1-phosphate uridylyltransferase | *Enterobacter cloacae* | Enterobacteria |
| **37** | CBG88927 | UTP-glucose-1-phosphate uridylyltransferase. Gene *galF* | *Citrobacter rodentium* ICC168 | Enterobacteria |
| **38** | CBG88529 | UTP-glucose-1-phosphate uridylyltransferase. Gene *galU* | *Citrobacter rodentium*ICC168 | Enterobacteria |
| **39** | CAQ89629 | putative subunit with GalU | *Escherichia fergusonii* ATCC 35469 | Enterobacteria |
| **40** | CAQ89235 | glucose-1-phosphate uridylyltransferase | *Escherichia fergusonii* ATCC 35469 | Enterobacteria |
| **41** | CBA21335 | UTP-glucose-1-phosphate uridylyltransferase. Gene *galF* | *Erwinia amylovora* CFBP1430 | Enterobacteria |
| **42** | CBA20912 | glucose-1-phosphate uridylyltransferase. Gene *galU* | *Erwinia amylovora* CFBP1430 | Enterobacteria |
| **43** | CAA09328 | UDP-glucose pyrophosphorylase [*Pseudomonas aeruginosa* PAO1] | *Pseudomonas aeruginosa* PAO1 | g-proteobacteria Pseudomonales |
| **44** | AHZ72597 | UDP-glucose pyrophosphorylase [*Pseudomonas mandelii* JR-1] | *Pseudomonas mandelii* JR-1 | g-proteobacteria Pseudomonales |
| **45** | BAO62770 | UDP-glucose pyrophosphorylase [*Pseudomonas protegens* Cab57] | *Pseudomonas protegens* Cab57 | g-proteobacteria Pseudomonales |
| **46** | AAB17376 | UDP-glucose pyrophosphorylase [*Xanthomonas campestris*] | *Xanthomonas campestris* | g-proteobacteria Xhantomonadaceae |
| **47** | AGI07748 | UDP-glucose pyrophosphorylase [*Xanthomonas citri* subsp. citri Aw12879] | *Xanthomonas citri* subsp. *citri* Aw12879 | g-proteobacteria Xhantomonadaceae |
| **48** | AGG88932 | UDP-glucose pyrophosphorylase [*Rhodanobacter denitrificans*] | *Rhodanobacter denitrificans* | g-proteobacteria Xhantomonadaceae |
| **49** | EGO82327 | UDP-glucose pyrophosphorylase [*Xylella fastidiosa* EB92.1] | *Xylella fastidiosa* EB92.1 | g-proteobacteria Xhantomonadaceae |
| **50** | ENZ93249 | UDP-glucose pyrophosphorylase [*Ruegeria mobilis* F1926] | *Ruegeria mobilis* F1926 | Rhodobacterales |
| **51** | EKE74477 | UDP-glucose pyrophosphorylase [*Celeribacter baekdonensis* B30] | *Celeribacter baekdonensis* B30 | Rhodobacterales |
| **52** | EGJ22660 | UDP-glucose pyrophosphorylase [*Rhodobacter sphaeroides* WS8N] | *Rhodobacter sphaeroides* WS8N | Rhodobacterales |
| **53** | EAU45350 | UDP-glucose pyrophosphorylase [*Pelagibaca bermudensis* HTCC2601] | *Pelagibaca bermudensis* HTCC2601 | Rhodobacterales |
| **54** | ABL70388 | UDP-glucose pyrophosphorylase [*Paracoccus denitrificans* PD1222] | *Paracoccus denitrificans* PD1222 | Rhodobacterales |
| **55** | KFZ75390 | UDP-glucose pyrophosphorylase [*Mycobacterium tuberculosis*] | *Mycobacterium tuberculosis* | Mycobacteriaceae |
| **56** | KFW57570 | UDP-glucose pyrophosphorylase [*Mycobacterium bovis*] | *Mycobacterium bovis* | Mycobacteriaceae |
| **57** | AHC27119 | UDP-glucose pyrophosphorylase [*Mycobacterium neoaurum* VKM Ac-1815D] | *Mycobacterium neoaurum* VKM Ac-1815D | Mycobacteriaceae |
| **58** | AEF41995 | UDP-glucose pyrophosphorylase [*Amycolicicoccus subflavus*] | *Amycolicicoccus subflavus* DQS3-9A1 | Mycobacteriaceae |
| **59** | AEI26273 | UDP-glucose pyrophosphorylase [*Streptomyces hygroscopicus* subsp. *jinggangensis*] | *Streptomyces hygroscopicus* subsp. *jinggangensis* | Streptomycetaceae |
| **60** | ADI09606 | UTP-glucose-1-phosphate uridylyltransferase [*Streptomyces bingchenggensis* BCW-1] | *Streptomyces bingchenggensis* BCW-1 | Streptomycetaceae |
| **61** | AAK34830 | UDP-glucose pyrophosphorylase [*Streptococcus pyogenes* M1 GAS] | *Streptococcus pyogenes* M1 GAS | Firmicutes |
| **62** | ADL07257 | UDP-glucose pyrophosphorylase [*Thermosediminibacter oceani* DSM 16646] | *Thermosediminibacter oceani* DSM 16646 | Firmicutes |
| **63** | AAA71967 | UDP-glucose pyrophosphorylase [*Bacillus subtilis* subsp. *subtilis* str. 168] | *Bacillus subtilis* subsp. *subtilis* str. 168 | Firmicutes |
| **64** | EOD03021 | UDP-glucose pyrophosphorylase [*Lactobacillus delbrueckii* ZN7a-9] | *Lactobacillus delbrueckii* ZN7a-9 | Firmicutes |
| **65** | ADM85669 | UDP-glucose pyrophosphorylase [*Streptococcus pneumoniae* AP200] | *Streptococcus pneumoniae* AP200 | Firmicutes |
